# HCC is associated with diabetes and longitudinal blood glucose control in a national cohort with cirrhosis

**DOI:** 10.1097/HC9.0000000000000344

**Published:** 2023-12-07

**Authors:** Catherine Mezzacappa, Nadim Mahmud, Marina Serper, Binu V. John, Tamar H. Taddei, David E. Kaplan

**Affiliations:** 1 Department of Internal Medicine, Section of Digestive Diseases, Yale School of Medicine, New Haven, Connecticut, USA; 2 VA Connecticut Healthcare System, Department of Internal Medicine West Haven, Connecticut, USA; 3 Division of Gastroenterology and Hepatology, Perelman School of Medicine, University of Pennsylvania, Philadelphia, Pennsylvania, USA; 4 Department of Medicine, Corporal Michael J. Crescenz VA Medical Center, Philadelphia, Pennsylvania, USA; 5 University of Miami School of Medicine, Miami, Florida, USA; 6 Bruce W Carter VA Medical Center, Miami, Florida, USA

## Abstract

**Background:**

Diabetes is associated with HCC; however, the impact of longitudinal blood glucose (BG) control on HCC risk in cirrhosis is not well known. We investigated this knowledge gap in a cohort of United States Veterans with cirrhosis from 2015 to 2021.

**Methods:**

We used repeated hemoglobin A1c measurements to categorize follow-up time according to BG control (defined as hemoglobin A1c < 7%) state over time: uncontrolled, nonsustained control (≤2 y), or sustained control (>2 y). We performed a sensitivity analysis using hemoglobin A1c < 8% to define BG control. We used Fine and Gray Cox proportional hazards regression with death and transplant as competing events to compare rates of incident HCC.

**Results:**

Our study included 81,907 individuals, 56.2% of whom had diabetes at baseline. There were 8,002 incident HCCs. The rate of HCC was 18% higher in diabetes (95% CI: 13% – 24%), and the relative increase in the rate of HCC varied by etiology of cirrhosis from nonsignificant (HCV) to an increase of 120% (HBV). Uncontrolled and nonsustained BG control was associated with 1.80 (95% CI: 1.70–1.91) and 2.34 (95% CI: 2.21–2.48) times the rate of HCC compared to sustained BG control, respectively. Using Hgb A1c < 8% to define BG control, HCC rates in uncontrolled and nonsustained BG control were 2.43 (2.28–2.58) and 2.23 (2.11–2.36) times that observed in sustained BG control.

**Conclusions:**

Associations between diabetes and HCC in cirrhosis vary according to the longitudinal BG control state. Inadequate BG control is consistently associated with a higher risk of HCC, and long-term BG control should be considered in comprehensive cirrhosis care.

## Graphical abstract

**Figure:**
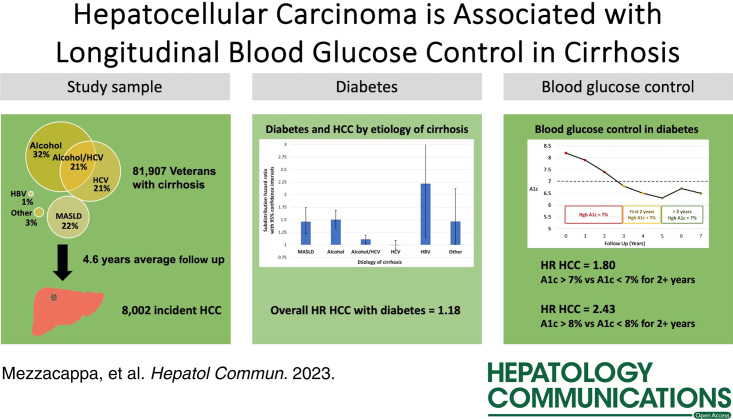

## INTRODUCTION

Diabetes is associated with an increased risk of multiple solid organ cancers, including HCC.^1–20^ Several biological mechanisms are thought to underly this association: the mitogenic effects of increased circulating insulin, increased bioavailable insulin-like growth factor 1, which promotes cellular proliferation and inhibits apoptosis, and direct effects of hyperglycemia on cancer cell behavior.^21–28^

The relationships between diabetes, chronic liver disease, and HCC are complex. Insulin resistance plays a central, bidirectional role in metabolic dysfunction–associated steatotic liver disease (MASLD, previously non-alcoholic fatty liver disease or NAFLD), which can lead to cirrhosis and HCC.^8,25,26,29^ Most HCC arises in persons with pre-existing cirrhosis, which can lead to hyperglycemia and increased risk of diabetes, but at advanced stages is associated with hypoglycemia. Observational studies have demonstrated that diabetes most often precedes incident HCC. This finding, along with dose-response relationships between the duration of diabetes, fasting blood glucose measurements, and incident HCC, support a potentially causal relationship.^3,8,11,14,18,30^ While the pathophysiological mechanisms underlying cancer risk in diabetes are thought to be driven by insulin and growth factor exposure, hemoglobin A1c (HgbA1c) measurements have been used as a proxy for insulin resistance and endogenous insulin exposure in clinical research.

**FIGURE 1:**
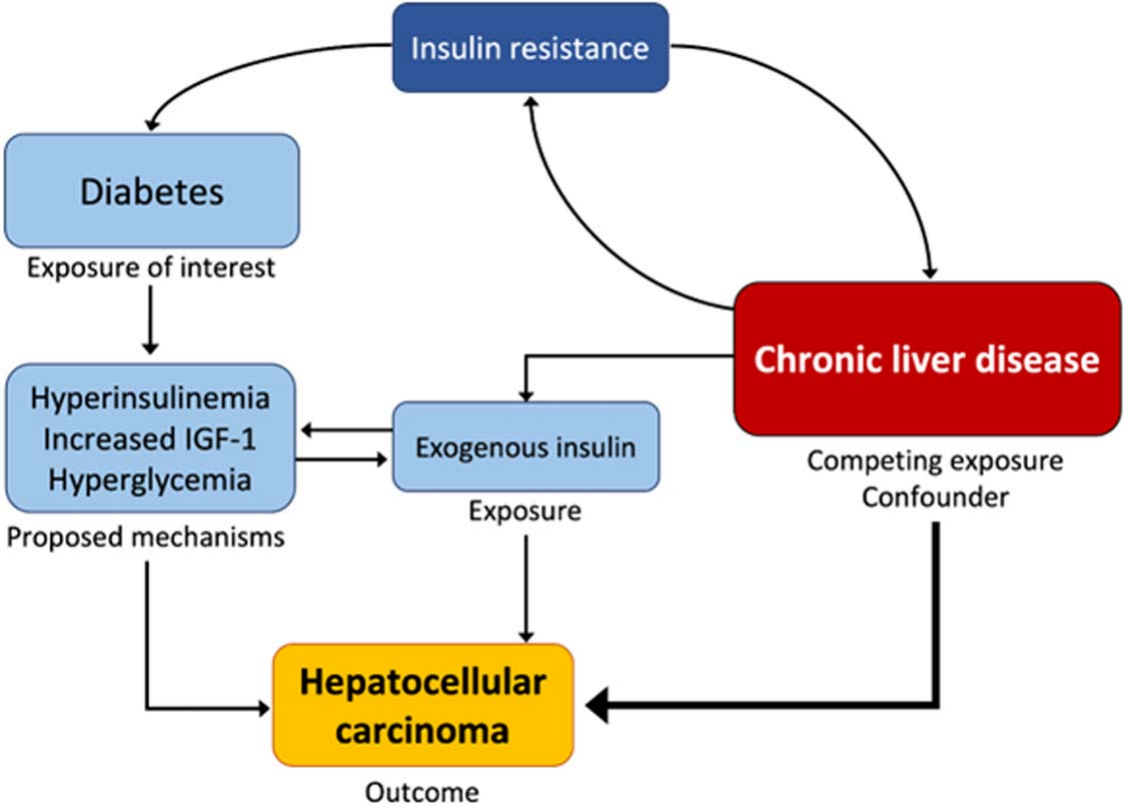
Associations between chronic liver disease, diabetes, and HCC. A directed acyclic graph demonstrates the proposed mechanisms underlying the association between diabetes and HCC on the left, the association between progressive chronic liver disease and HCC on the right, and the bidirectional interactions between the exposure of interest (diabetes) and the competing exposure and confounder (chronic liver disease). Abbreviation: IGF-1, insulin-like growth factor 1.

In cirrhosis, the presence of advanced chronic liver disease remains the main risk factor for HCC. Evaluating the association between diabetes, blood glucose control, and HCC risk in cirrhosis is complicated by the bidirectional relationships between the progression of hepatic and peripheral insulin resistance, chronic liver disease, blood glucose regulation, and physician selection of diabetes medications (Figure 1). Better understanding of the association between blood glucose control and HCC risk may change practices around blood glucose management in cirrhosis and inform how patients at risk of HCC are counseled regarding risk modification, in particular as novel anti-diabetic medications continue to emerge. This study evaluates the association between diabetes, longitudinal blood glucose control state, and HCC in a large cohort of Veterans with cirrhosis largely due to alcohol, HCV, and MASLD.

We evaluated the following hypotheses: (1a) diabetes is associated with increased rates of HCC in cirrhosis, and (1b) the relative increase in the rate of HCC associated with diabetes varies by etiology of underlying liver disease; (2a) in persons with cirrhosis and diabetes, inadequate blood glucose control is associated with a higher rate of HCC compared to adequate blood glucose control, and (2b) this association follows a dose-response relationship by duration of time with inadequate blood glucose control; (3) in persons with cirrhosis and diabetes, the association between blood glucose control and HCC is modified by underlying hepatic synthetic function.

## METHODS

This is a retrospective cohort study using data from a national cohort of adults with cirrhosis cared for in the VA Health System between January 1, 2014 and December 12, 2021. The development of this cohort has been described previously in detail.^31^ However, in brief, patients with cirrhosis were identified using a combination of either 1 inpatient or 2 outpatient ICD codes. Any ICD9 code for cirrhosis has a positive predictive value of 90% and a negative predictive value of 87% in Veterans Health Administration (VHA) data.^32^ The baseline period was defined as January 1, 2014 for patients with prevalent cirrhosis or as the date of cirrhosis diagnosis for additional incident diagnoses after this date. We excluded patients with a diagnosis of HCC at baseline or within 12 months after baseline to further exclude the possibility of pre-existing HCC at the time of enrollment. We also excluded patients with less than 1 year of follow-up from the baseline date. Patients included in the analytic cohort were observed for incident HCC beginning 1 year from baseline (index date, earliest January 1, 2015) to December 31, 2021, which was the maximum period of follow-up.

### Exposures

The primary exposure of interest was diabetes, which was defined based on a combination of diagnostic codes, HgbA1c measurements, blood glucose measurements, and diabetes medication prescriptions shown in Table 1 using a method developed and validated in VHA data.^33^ This composite definition carries a sensitivity of 95%, specificity of 94%, and positive predictive value of 70%.^33^ Date of diabetes diagnosis was the first date that the criteria were met. Individuals diagnosed with diabetes within the first 90 days after baseline were categorized as having diabetes at baseline. Time at risk for HCC was categorized as with diabetes or without diabetes, and Veterans who developed diabetes after baseline contributed follow-up time to both exposure states. Among those with diabetes, the duration of diabetes was calculated from the first date of diagnosis to the end of time at risk of HCC.

**TABLE 1:** Criteria for diagnosis of diabetes

Diabetes present if ≥ 1 of the following criteria is met:
(1) HgbA1c > 6.5% on 2 separate occasions (any duration apart) (2) HgbA1c > 6.5% on 1 occasion and treatment with an outpatient oral hypoglycemic or insulin > 30 d (3) ICD9/10 codes (2 outpatient or 1 inpatient) and treatment with an oral hypoglycemic or insulin for > 30 d (4) ICD9/10 codes (2 outpatient or 1 inpatient) and serum glucose > 126 mg/dL on 2 separate occasions (5) Serum glucose > 200 mg/dL on 2 separate occasions (any duration apart) (6) Serum glucose > 200 mg/dL on 1 occasion and treatment with an outpatient oral hypoglycemic or insulin for > 30 d

*Notes:* Adapted from McGinnis KA, Justice AC, Bailin S, Wellons M, Freiberg M, Koethe JR.^33^ Adaptations are themselves works protected by copyright. So to publish this adaptation, authorization must be obtained both from the owner of the copyright in the original work and from the owner of copyright in the translation or adaptation.

Abbreviations: HgbA1c, hemoglobin A1c; ICD, International Classification of Diseases.

In patients with diabetes, blood glucose control over time was ascertained using serial HgbA1c measurements, which were carried backward and forward in time until either (1) halfway to the next closest HgbA1c measurement or (2) 180 days, whichever came first. Adequate blood glucose control was defined as HgbA1c < 7% per ADA standards for non-pregnant adults for the primary analysis.^34^ All follow-up time was categorized as with or without adequate blood glucose control and the percent of time without adequate blood glucose control was calculated for all persons with diabetes. To evaluate the association between duration of blood glucose control and HCC, follow-up time was further categorized as without adequate blood glucose control, with nonsustained blood glucose control (within first 2 y of HgbA1c < 7%), or with sustained blood glucose control (HgbA1c < 7% for greater than 2 y). Two sets of sensitivity analyses were performed: (1) excluding individuals with diabetes with < 50% of follow-up time captured by available HgBA1c measurements, and (2) utilizing a HgbA1c threshold of 8% to define blood glucose control. The second sensitivity analysis was performed because many persons with cirrhosis would meet the criteria for clinical recommendations to target a more liberal HgbA1c goal between 7% and 9% based on limited life expectancy or significant comorbid disease.^35^

Additional covariables of interest were collected for each patient, including demographic data (age, sex, race, Hispanic ethnicity), body mass index, smoking status (never, current, prior), risky alcohol use as measured by the Alcohol Use Disorders Identification Test-Consumption,^36^ and comorbidities as measured by the CirCom score.^37^ The etiology of liver disease was ascertained using a previously validated VHA algorithm,^38^ and categorized as NAFLD, alcohol, mixed alcohol/HCV, HCV, HBV, or other etiology. Cirrhosis attributed to NAFLD was identified by excluding individuals with alcohol-associated diagnoses and requiring the presence of either diabetes or body mass index ≥30 prior to the diagnosis of cirrhosis, and individuals with NAFLD cirrhosis were considered to have MASLD cirrhosis in this study. Other etiology included autoimmune hepatitis (n = 190), primary biliary cholangitis (n = 133), primary sclerosing cholangitis (n = 189), hereditary hemochromatosis (n = 123), and cryptogenic cirrhosis (n = 1597). Among Veterans with a history of HCV, all HCV RNA measurements and outpatient prescriptions for direct-acting antivirals (DAAs) for HCV were used to categorize individuals as with sustained virologic response (SVR) by DAA, SVR but not treated with DAA, HCV not treated with DAA and not cleared, or HCV treated with DAA and not cleared. Child-Turcotte-Pugh (CTP) class was determined using a validated VHA algorithm^39^ at baseline and matched to blood glucose control periods during follow-up. Outpatient primary care, gastroenterology, and hepatology visits were tabulated and used to calculate the annual number of visits during follow-up.

### Outcomes

The primary outcome was time to incident diagnosis of HCC, which was defined as 1 inpatient or 2 outpatient International Classification of Diseases (ICD), 9th and 10th Revision, Clinical Modification (ICD9/10-CM) primary or secondary diagnosis codes for malignant neoplasm of the liver/liver cell carcinoma (ICD9-CM: 155.0, 155.2; ICD10-CM: C22.0) with no ICD9/10 codes for cholangiocarcinoma (ICD9-CM: 155.1; ICD10-CM: C22.1). These codes have previously been validated in VHA data with a positive predictive value of 78.2% for confirmed HCC.^40^ As noted above, patients with HCC at baseline or within 12 months of baseline were excluded to avoid capturing prevalent HCC. Deaths were ascertained using the Vital Status Master File, and liver transplants were identified by cross-referencing the UNOS database.

### Primary statistical analyses

Descriptive statistics stratified by diabetes status were presented as medians with interquartile ranges or means with SDs for continuous variables based on observed distributions and as frequencies and percentages for categorical variables. Statistical comparisons were made using Wilcoxon rank-sum, *t*-test, or chi-square tests as indicated. Overall cumulative incidence, incidence rates, and HCC-free survival were calculated in veterans with and without diabetes. A Fine and Gray Cox proportional hazards regression was then used to compare the relative difference in the rate of incident HCC, represented by the subdistribution HR (SHR), by time-updating diabetes status adjusted for potential confounders, treating death and liver transplant as competing events. Time zero was 1 year after the baseline date, and data were right-censored at loss-to-follow-up or maximum follow-up. All models were adjusted for the following *a priori* baseline covariables (except when used as a stratifying variable): age, race/ethnicity, body mass index, etiology of cirrhosis, CTP class, smoking status (never, former, current), Alcohol Use Disorders Identification Test-Consumption, HCV treatment status, statin use, and annual visit frequency with primary care and gastroenterology or hepatology.

### Diabetes subgroup analysis

A second proportional hazards regression was performed restricted to all Veterans with diabetes at baseline to evaluate the duration of diabetes (y), percent of follow-up time without adequate blood glucose control, and insulin exposure as predictors of HCC among those with cirrhosis and diabetes. Models were additionally adjusted for the covariables listed above. Finally, proportional hazards regression with time-updating repeated measurements was used to evaluate the association between blood glucose control state (inadequate blood glucose control, nonsustained blood glucose control, sustained blood glucose control) and rate of incident HCC. This model adjusted for time-updating CTP class and duration of diabetes corresponding to the window of blood glucose control state in addition to the other covariables previously listed.

### Sensitivity analyses

Measurement of HgbA1c may be associated with severity of underlying diabetes. We therefore evaluated the proportion of follow-up time represented by a HgbA1c value for all individuals with diabetes at baseline and performed a sensitivity analysis in which individuals with less than 50% of follow-up were captured by a HgbA1c measurement. We also compared the average HgbA1c value during follow-up for those with 50% or more of follow-up time captured by a HgbA1c value to those with less than 50% of follow-up time captured. The results of this sensitivity analysis for the total sample and for the diabetes subgroup are presented in Supplemental Table S2, http://links.lww.com/HC9/A701.

Diagnosis of HCC is typically made using imaging. To address the potential differential likelihood of HCC diagnosis by imaging receipt, we identified all abdominal ultrasounds and contrast-enhanced abdominal CT and MRI studies obtained for individuals in our sample. We performed a sensitivity analysis excluding all individuals without one of the above imaging studies within 2 years of the end of follow-up. The results of this sensitivity analysis for the total sample and for the diabetes subgroup are presented in Supplemental Tables S3, http://links.lww.com/HC9/A701.

### Ethical considerations and other software details

This study received Institutional Review Board approval from the Michael J. Crescenz Philadelphia Veterans Affairs Medical Center. All data management and analyses were performed using SAS Enterprise Guide 8.3 (Cary, NC, USA).

## RESULTS

### Cohort characteristics

A total of 81,907 veterans with cirrhosis, 56.2% of whom had diabetes at baseline and 1.9% of whom developed diabetes after cirrhosis, were included. The most common causes of cirrhosis in the sample were alcohol (32.1%), mixed alcohol/HCV (21.4%), HCV alone (20.9%), and MASLD (21.9%). Prevalence of diabetes varied by etiology of cirrhosis from 51.2% (mixed alcohol/HCV) to 76.0% (MASLD) (Supplemental Table S1, http://links.lww.com/HC9/A701). The average age in the sample was 63.0 years (SD 8.9 y) at baseline, and the sample was almost entirely (97.2%) male. The majority (61.1%) of veterans included were White, 18.8% were Black, 8.4% were Hispanic, 1.2% were Asian or Native American, and 9.2% identified as another race. Veterans who developed diabetes during follow-up were younger and more likely to have CTP A cirrhosis at baseline (Table 2). Among individuals with diabetes, the median number of A1c measurements during follow-up was 7 (interquartile range 3–12). The median percent of follow-up time with a corresponding HgbA1c was 93.0% (interquartile range 68.5%–100.0%) using the method described above. The distribution of HgbA1c measurements during follow-up is shown in Supplemental Figure S1, http://links.lww.com/HC9/A702.

**TABLE 2:** Sample characteristics by the presence of diabetes

	Total n = 81,907, %	No diabetes n = 34,314, %	Diabetes at baseline n = 46,035, %	Diabetes during follow-up n = 1558, %	*p*
Age (y)	63.0 (8.9)	62.0 (9.0)	63.9 (8.7)	58.6 (8.1)	<0.001
Male sex	97.2	97.0	97.3	97.1	0.02
Race and ethnicity					<0.001
White	61.1	62.1	60.4	60.6	
Black	18.8	18.6	18.9	18.7	
Hispanic	8.4	7.4	9.2	8.5	
Asian	1.2	1.2	1.3	1.5	
Native American	1.2	1.2	1.2	2.2	
Other	3.8	4.2	3.6	2.6	
Unknown	5.4	5.4	5.4	5.9	
Etiology of cirrhosis					<0.001
Alcohol	32.1	34.4	30.3	36.3	
Alcohol/HCV	21.4	24.9	18.8	24.7	
HCV	20.9	23.8	18.7	20.5	
MASLD	21.9	12.5	29.2	13.0	
HBV	0.9	1.0	0.9	0.8	
Other	2.7	3.4	2.1	4.8	
Unknown	0.1	0.1	0.0	0.0	
HCV status					<0.001
Never HCV-infected	57.7	51.3	62.5	54.9	
Cleared HCV	26.1	31.2	22.0	33.3	
Persistent HCV	16.3	17.5	15.5	11.9	
Unknown	0.1	0.1	0.0	0.0	
AUDIT-C					<0.001
Low risk	71.0	63.6	76.7	64.9	
Moderate risk	9.5	11.9	7.6	10.5	
High risk	4.2	5.4	3.3	6.1	
Severe risk	11.9	15.7	9.0	15.7	
Unknown	3.4	3.5	3.4	3.1	
CTP class					<0.001
A	61.1	44.9	70.4	79.3	
B	32.3	44.7	26.1	18.1	
C	6.6	10.4	3.5	2.7	
Statin use	38.1	23.0	50.0	20.2	<0.001
Years of follow-up	4.6 (3.4)	4.8 (3.5)	4.5 (3.4)	6.7 (3.7)	<0.001
HCC incidence (per 100 person-years)	2.11	1.96	2.18	2.99	<0.001
Incident HCC	9.8	9.3	9.8	20.2	<0.001
Deceased	50.3	46.0	54.1	32.2	<0.001

*Note*: Continuous variables age and years of follow-up are presented as mean (SD).

Abbreviations: AUDIT-C, Alcohol Use Disorders Identification Test-Consumption; CTP Child-Turcotte-Pugh; MASLD, metabolic dysfunction–associated steatotic liver disease.

### Association between diabetes and incident HCC

**FIGURE 2:**
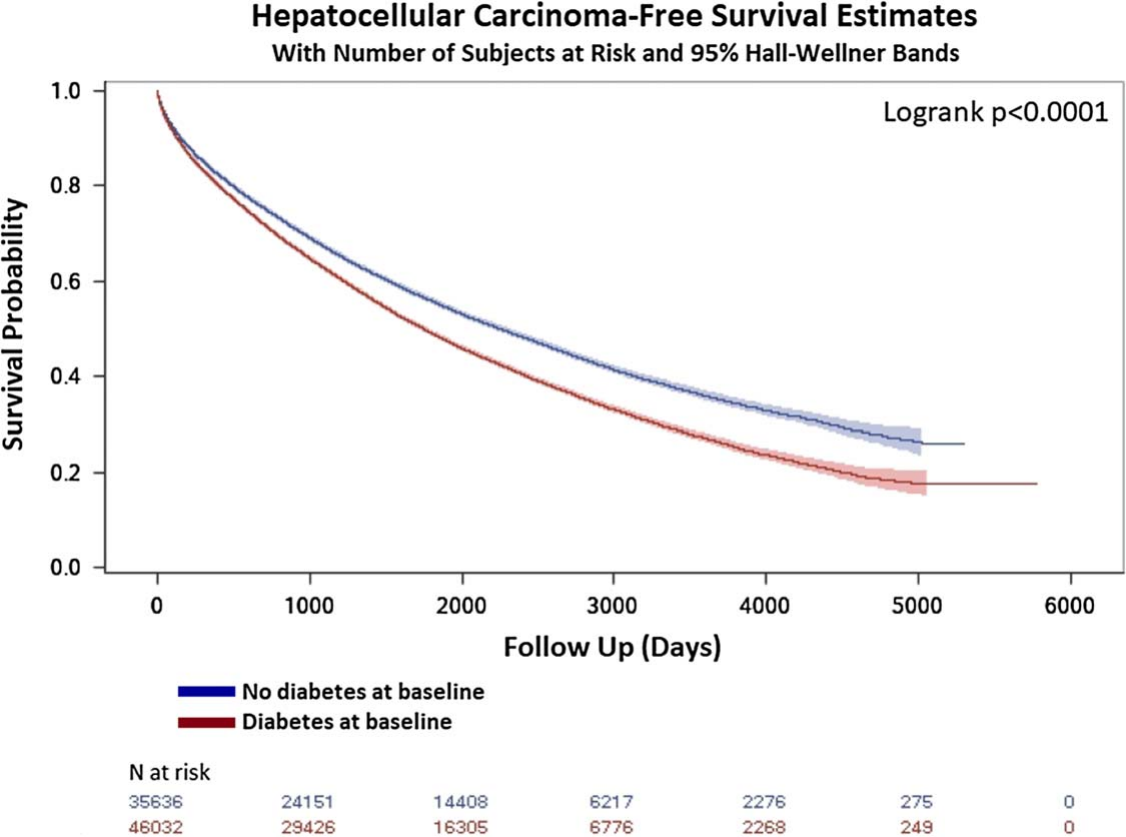
Unadjusted Kaplan-Meier survival function by diabetes diagnosis. HCC-free survival function stratified by baseline diabetes status.

Over a mean follow-up of 4.6 years (SD=3.4 y), 3,187 patients without diabetes (9.29%), 4,501 patients with DM at baseline (9.8%), and 314 patients with new DM after cirrhosis (20.2%) were diagnosed with incident HCC. There was a significant difference in HCC-free survival in unadjusted Kaplan-Meier survival analysis, whereby patients with prevalent diabetes were more likely to develop HCC (Figure 2).

**FIGURE 3:**
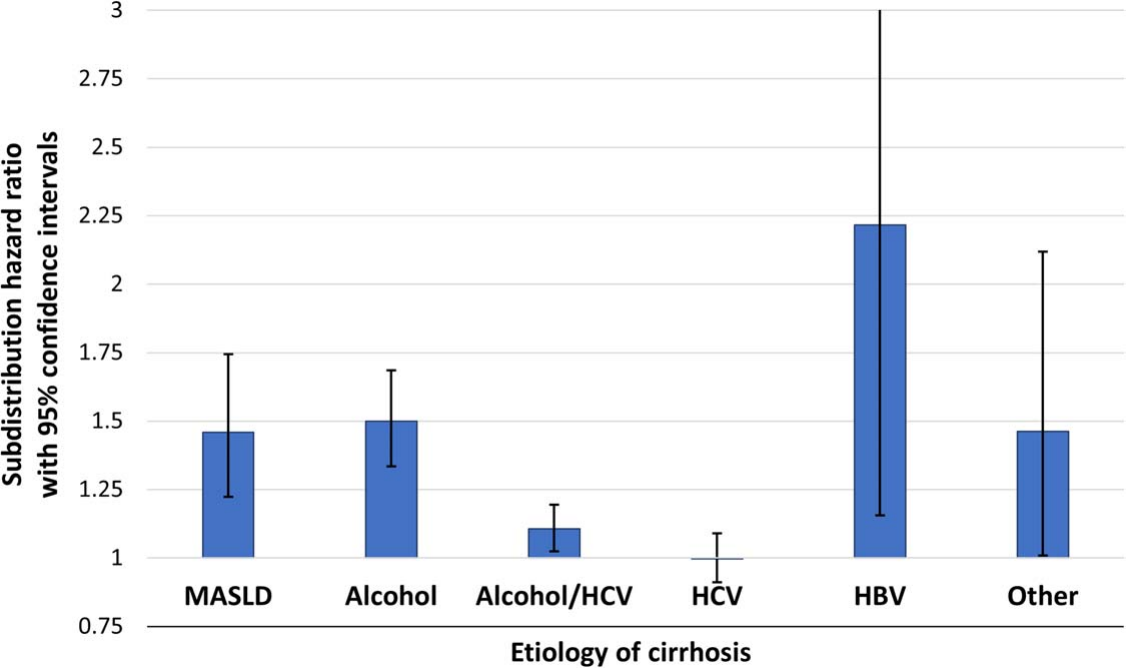
Association between diabetes status and HCC by etiology of cirrhosis. SHRs from Cox proportional hazards regression model testing for interaction between diabetes and etiology of cirrhosis adjusted for HCV treatment status, CTP class, AUDIT-C, age, sex, race/ethnicity, BMI, tobacco use, annual primary care visits, and annual gastroenterology/hepatology visits. Abbreviations: AUDIT-C, Alcohol Use Disorders Identification Test-Consumption; CTP Child-Turcotte-Pugh; MASLD, metabolic dysfunction–associated steatotic liver disease; SHR, Subdistribution HR.

In adjusted models, we found 18% higher rate of HCC in diabetes (SHR = 1.18, 95% CI: 1.13–1.24) (Table 3). There was a significant statistical interaction between diabetes and the etiology of cirrhosis (*p* < 0.001). Stratified by etiology of cirrhosis, the strongest association between diabetes and HCC was in persons with HBV cirrhosis (SHR=2.22, 95% CI: 1.16–4.25), followed by alcohol (SHR = 1.50, 95% 1.33–1.69), then MASLD (SHR=1.46, 95% CI: 1.22–1.74). There was a borderline association in those with mixed alcohol and HCV cirrhosis (SHR = 1.11, 95% CI: 1.02–1.19) and no association between diabetes and HCC in those with cirrhosis due to HCV alone (Figure 3). In the sensitivity analysis excluding those with diabetes and less than 50% of follow-up time captured by HgbA1c measurements, we observed a slightly larger relative increase in HCC rate associated with diabetes (SHR = 1.24, 95% CI: 1.17–1.30; full model results in Supplemental Table S2, http://links.lww.com/HC9/A701). In the sensitivity analysis excluding individuals without abdominal imaging during the last 2 years of follow-up, the association between diabetes and HCC was slightly attenuated (SHR = 1.14, 95% CI: 1.08–1.19; full model results in Supplemental Table S3, http://links.lww.com/HC9/A701).

**TABLE 3:** Association of diabetes status with HCC

Predictor	SHR	95% CI
Diabetes	1.18	1.13–1.24
Etiology of cirrhosis		
MASLD	Ref	—
Alcohol	1.22	1.12–1.34
Alcohol/HCV	2.94	2.52–3.44
HCV	2.94	2.54–3.42
HBV	1.47	1.14–1.88
Other	1.06	0.88–1.27
HCV treatment status		
Non-HCV cirrhosis	Ref	—
HCV treated by DAA, cleared	0.13	0.11–0.15
HCV cleared (non-DAA)	0.05	0.03–0.07
HCV treated by DAA, not cleared	No est	No est
HCV not treated, not cleared	2.34	2.04–2.67
Baseline CTP class		
A	Ref	—
B	0.59	0.56–0.63
C	0.46	0.40–0.53
Statin use	0.69	0.65–0.73
AUDIT-C		
Low risk	Ref	—
Moderate risk	0.95	0.87–1.04
High risk	0.90	0.80–1.01
Severe risk	0.76	0.70–0.83
Age (per y)	1.01	1.01–1.01
Male sex	1.83	1.51–2.21
Race/ethnicity		
Non-Hispanic White	Ref	—
Black	0.94	0.89–1.00
Asian	1.23	1.02–1.48
Hispanic	1.04	0.96–1.13
Native American	1.12	0.92–1.37
Other	0.84	0.77–0.91
BMI (per point increase)	1.01	1.01–1.01
Tobacco use		
Never	Ref	—
Former	1.05	0.99–1.11
Current	1.00	0.94–1.06
Annual primary care visits	0.99	0.98–1.00
Annual GI/hepatology visits	1.02	1.01–1.03

*Note*: Missing data for covariables was present as follows: HCV treatment status, n = 38, AUDIT-C, n = 291, smoking status, n = 1136, BMI n = 292.

Abbreviations: AUDIT-C, Alcohol Use Disorders Identification Test-Consumption; BMI, body mass index; CTP Child-Turcotte-Pugh; DAA, direct-acting antivirals; MASLD, metabolic dysfunction–associated steatotic liver disease; SHR, subdistribution HR.

### Diabetes subgroup analysis evaluating the association of blood glucose control and HCC

Among those with diabetes, a more recent diagnosis of diabetes was associated with a higher relative rate of incident HCC: those diagnosed with diabetes within the 2 years prior to being diagnosed with cirrhosis had a 34% higher rate of HCC than those diagnosed with diabetes 10 or more years before cirrhosis (SHR = 1.34, 95% 1.22–1.48) and those diagnosed with diabetes between 2 and 5 years before cirrhosis had 15% greater HCC rate (SHR = 1.147, 95% CI: 1.04–1.27). Treatment with insulin for 5 or more years prior to baseline was associated with a lower HCC rate (SHR = 0.83, 95% CI: 0.73–0.94). Every 10% increment of follow-up time with uncontrolled blood glucose (HgbA1c > 7%) was associated with a 3% increased rate of HCC (SHR = 1.03, 95% CI: 1.02–1.04) (Table 4). The results of sensitivity analyses restricted to individuals with at least 50% of follow-up represented by HgbA1c measurements and with abdominal imaging within 2 years of the end of follow-up were similar. Model results are shown in Supplemental Tables S2, http://links.lww.com/HC9/A701 and S3, http://links.lww.com/HC9/A701, respectively.

**TABLE 4:** Factors associated with HCC in persons with cirrhosis and diabetes^a^

Predictor	SHR	95% CI
Duration of diabetes		
10+ y before cirrhosis	Ref	—
5–10 y before cirrhosis	1.08	1.00–1.18
2–5 y before cirrhosis	1.15	1.04–1.27
0–2 y before cirrhosis	1.34	1.22–1.48
Insulin exposure at baseline		
No insulin exposure	Ref	—
5+ y before cirrhosis	0.83	0.73–0.94
2–5 y before cirrhosis	0.90	0.79–1.02
0–2 y before cirrhosis	0.83	0.75–1.04
Percent of follow-up with HgbA1c > 7% (per 10% increase)	1.03	1.02–1.04

*Note*: Missing data for covariables was present as follows: AUDIT-C n = 124, smoking status n = 592, BMI n = 77.

a Model further adjusted for HCV treatment status, baseline CTP class, statin use, AUDIT-C, age, sex, race/ethnicity, BMI, and tobacco use.

Abbreviations: AUDIT-C, Alcohol Use Disorders Identification Test-Consumption; BMI, body mass index; CTP Child-Turcotte-Pugh; HgbA1c, hemoglobin A1c; SHR, subdistribution HR.

In the proportional hazards regression analysis using repeated measures of blood glucose control state, the nonsustained blood glucose control and uncontrolled blood glucose states were associated with 2.34 (95% CI: 2.21–2.48) and 1.80 (95% CI: 1.70–1.91) times the rate of HCC compared to sustained blood glucose control, respectively. Stratified by CTP class matched to the window of blood glucose control, the effect sizes were slightly attenuated in CTP C cirrhosis: nonsustained blood glucose control was associated with 2.40 (95% CI: 2.24–2.56), 2.27 (95% CI: 2.00–2.57), and 1.75 (95% CI: 1.25–2.45) times the rate of HCC in CTP A, B and C cirrhosis and uncontrolled blood glucose state was associated with 1.76 (95% CI: 1.65–1.88), 2.00 (95% CI: 1.76–2.28), and 1.93 (95% CI: 1.28–2.92) times the rate of HCC (Table 5).

**TABLE 5:** Risk of HCC in persons with cirrhosis and diabetes by time-updating blood glucose control state, total sample, and stratified by CTP class

	Main analysis^a^: Blood glucose control defined as HgbA1c < 7%			Sensitivity analysis^a^: Blood glucose control defined as HgbA1c < 8%		
		SHR	95% CI	SHR	95% CI	
Total Sample	Sustained BG control (≥2 y)	Ref	—	Sustained BG control (≥2 y)	Ref	—
	Nonsustained BG control (0–2 y)	2.34	2.21–2.48	Nonsustained BG control (0–2 y)	2.23	2.11–2.36
	Uncontrolled BG	1.80	1.70–1.91	Uncontrolled BG	2.43	2.28–2.58
CTP A	Sustained BG control (≥2 y)	Ref	—	Sustained BG control (≥2 y)	Ref	—
	Nonsustained BG control (0–2 y)	2.40	2.24–2.56	Nonsustained BG control (0–2 y)	2.31	2.17–2.47
	Uncontrolled BG	1.76	1.65–1.88	Uncontrolled BG	2.41	2.25–2.59
CTP B	Sustained BG control (≥2 y)	Ref	—	Sustained BG control (≥2 y)	Ref	—
	Nonsustained BG control (0–2 y)	2.27	2.00–2.57	Nonsustained BG control (0–2 y)	2.06	1.83–2.33
	Uncontrolled BG	2.00	1.76–2.28	Uncontrolled BG	2.60	2.27–2.99
CTP C	Sustained BG control (≥2 y)	Ref	—	Sustained BG control (≥2 y)	Ref	—
	Nonsustained BG control (0–2 y)	1.75	1.25–2.45	Nonsustained BG control (0–2 y)	1.95	1.42–2.69
	Uncontrolled BG	1.93	1.28–2.92	Uncontrolled BG	3.04	1.94–4.76

a Models adjusted for time-updating CTP class except where stratified by CTP class matched to the time period of blood glucose control status, baseline statin use, HCV treatment status, AUDIT-C, age, sex, race/ethnicity, BMI, tobacco use.

Abbreviations: AUDIT-C, Alcohol Use Disorders Identification Test-Consumption; BG, blood glucose; BMI, body mass index; CTP Child-Turcotte-Pugh; HgbA1c, hemoglobin A1c; SHR, subdistribution HR.

In sensitivity analyses using a threshold for blood glucose control of HgbA1c < 8%, nonsustained blood glucose control was associated with 2.23 times the rate of HCC (95% CI: 2.11–2.36) compared to sustained blood glucose control, while uncontrolled blood glucose was associated with 2.43 times the rate (95% CI: 2.28–2.58). Stratified by CTP class, the relative increases in HCC rates observed in CTP A cirrhosis were similar to that in the total sample: nonsustained blood glucose control SHR = 2.31 (95% CI: 2.17–2.47), uncontrolled blood glucose control SHR = 2.41 (95% CI: 2.25–2.59) and the associations between blood glucose control state and rate of HCC persisted in CTP B and CTP C cirrhosis (Table 5).

## DISCUSSION

We found that diabetes, as well as inadequate blood glucose control and initiation of insulin among those with diabetes, were independently associated with an increased rate of HCC among Veterans with existing cirrhosis. We carefully characterized diabetes, longitudinal blood glucose control, and hepatic function in this large, multi-center cohort of individuals with cirrhosis from a variety of underlying causes, and these results add to our understanding of the complex interactions between advanced chronic liver disease, diabetes, and HCC risk. In this study, we build upon previous findings that support the working hypothesis that the trophic effects of insulin (endogenous or exogenous) lead to an increased risk of HCC in diabetes^21–30,40,41^ in a cohort of persons with cirrhosis.

### Diabetes, blood glucose control, and HCC

Three recent observational studies have evaluated the association between blood glucose control and HCC risk in cohorts representing either a single etiology of liver disease or diabetes alone. Kramer et al^20^ found that in a cohort of Veterans with diabetes and MASLD, but not necessarily cirrhosis, adequate blood glucose control (HgbA1c < 7%) for 80% or more of follow-up time was associated with decreased risk of HCC (HR = 0.68). Evaluating a cohort of Chinese patients with chronic HBV, about 10% of whom had F4 fibrosis at baseline, Mak et al^41^ found that longer duration of diabetes and higher average HgbA1c were independently associated with HCC risk. In a registry of persons with diabetes, but not necessarily liver disease, Mao et al^42^ found that a higher glycemic burden measured over time was associated with an increased risk of HCC. Our work adds to recent literature by evaluating blood glucose control in a cohort with established cirrhosis due to diverse underlying liver diseases.

In their study, which also used VHA data, Kramer et al^20^ used repeated HgbA1c measurements and the standard definition of HgbA1c < 7% for blood glucose control. Their sample included Veterans with MASLD with a mean fibrosis-4 in the study sample of 1.3 (SD 0.99). The strongest single predictor of HCC in their sample was an fibrosis-4 ≥ 1.45 (ie, those who did not rule out advanced fibrosis). Our results build upon their findings by evaluating the relationships between diabetes, blood glucose control, insulin exposure, and HCC in the presence of existing advanced chronic liver disease, which remains the primary risk factor for HCC. Given the high baseline risk of HCC in cirrhosis, in this sample, the effect associated with diabetes is attenuated when compared to that measured in populations without cirrhosis. However, our findings demonstrate that careful diabetes management can contribute to comprehensive HCC risk reduction in this at-risk population.

We found that diabetes was associated with a higher rate of HCC in veterans with HBV, ASLD, and MASLD cirrhosis. This is consistent with previously published observations in cohorts with MASLD and HBV.^1–4,11,14,20,41,43^ Our finding that diabetes was not associated with increased HCC rates after accounting for HCV treatment status in this large (n = 17,388) sample of persons with HCV cirrhosis and diabetes supports results recently published by Tsai et al,^44^ who found an increased risk of HCC associated with diabetes among persons with HCV after SVR, but no difference in HCC risk in a sub-sample of 568 individuals with HCV-associated cirrhosis after SVR.

We also found that among those with alcohol-associated cirrhosis, diabetes was associated with a relative increase in the rate of HCC, similar to that observed in MASLD cirrhosis (alcohol SHR = 1.48, MASLD SHR = 1.43). About one-third of veterans with alcohol-associated cirrhosis had diabetes at baseline and may have had MetALD. Validation studies assessing the timing of steatosis onset, metabolic risk factors, and alcohol use are needed to identify MetALD in administrative datasets. Nonetheless, this finding adds to the growing recognition of mechanistic overlap between alcohol-associated and MASLD-associated chronic liver disease and outcomes in the context of parallel epidemics of alcohol use and metabolic disease.

Compared to those who had diabetes at baseline, those who developed diabetes after cirrhosis were younger, less likely to have MASLD cirrhosis, and more likely to develop HCC (20.2% vs. 9.8% cumulative incidence, 3 vs. 2.2 cases per 100 person-y). These individuals who developed diabetes were also more likely to have CTP A cirrhosis at baseline than those who did not develop diabetes (79.3% vs. 44.9%), so they may also be more likely to benefit from screening and diagnosis of early-stage HCC amenable to curative therapies. Clinicians caring for individuals with cirrhosis should ensure adequate HCC screening among those with recently diagnosed or new diabetes.

Among those with cirrhosis and diabetes at baseline, we observed a higher rate of incident HCC among those who initiated insulin after developing cirrhosis. Unexpectedly, longstanding (> 5 y) insulin use was associated with a lower HCC rate in this sample (Table 4). Both endogenous insulin bioavailability and exogenous insulin therapy are associated with an increased risk of HCC.^3,4,11–13,15,18,20,43,45^ The association between the duration of exogenous insulin therapy and HCC may be confounded by the control of hyperglycemia and endogenous insulin bioavailability, which are represented in part by the blood glucose control state. Multiple noninsulin therapies, for example the, glucagon-like peptide-1 agonists, are increasing in use and clinicians and patients may select from a variety of noninsulin therapies to achieve blood glucose control.

When we liberalized the definition of blood glucose control to HgbA1c < 8%, the relative increases in the rate of HCC associated with nonsustained blood glucose control and uncontrolled blood glucose were larger than using the HgbA1c < 7% threshold. This finding suggests that the standard threshold for blood glucose control in diabetes may not be the most biologically relevant threshold when assessing the risk of HCC in cirrhosis, a finding that is relevant in this population who may be advised to target a higher HgbA1c goal because of comorbid liver disease.

Taken together, these findings support optimizing blood glucose control in persons with cirrhosis and diabetes as part of comprehensive efforts at HCC risk reduction alongside minimizing hepatic inflammation from viral disease, MASLD, alcohol, and other insults. In the context of established associations between exogenous insulin treatment and cancer, using insulin to achieve blood glucose control may balance the risk of HCC in persons with cirrhosis. Targeting a less stringent HgbA1c goal of < 8% may be adequate to reduce HCC risk associated with diabetes but requires further study.

### Chronic liver disease and HCC risk in diabetes

In cirrhosis, changes in HgbA1c may reflect the progression of underlying liver disease, in which average blood glucose declines because of impaired hepatic gluconeogenesis. In a prior study of metformin use and overall survival performed in the same cohort of veterans with cirrhosis, low HgbA1c values were associated with an increased relative risk of all-cause mortality, a relationship that is thought to reflect the decline in hepatic functional reserve^46^ (Supplemental Figure 1, http://links.lww.com/HC9/A702). When we stratified by the CTP class matched to each window of blood glucose control, the association between uncontrolled blood glucose and HCC was attenuated in CTP C cirrhosis. These observations suggest that either the effect of diabetes on HCC risk occurs through influencing the histologic progression of liver disease (and therefore after progression, the effect is no longer observed), or that the relationship between blood glucose control and HCC risk in cirrhosis is modified by degree of underlying hepatic dysfunction. The dynamic nature of this relationship underscores the importance of accounting for progression of chronic liver disease when studying diabetes and HCC.^47^

We observed fewer overall HCC events in veterans with CTP C cirrhosis, which is attributable to the increased risk of all-cause mortality in these patients and the competing risk of death. Persons who died (without developing HCC) during follow-up were also older, more likely to have diabetes, and more likely to have alcohol-associated cirrhosis (Supplemental Table S4, http://links.lww.com/HC9/A701). Since our models compared HCC event rates (and not cumulative incidence), differential progression to death without HCC should not bias the evaluation of the associations of interest.^47^

### Limitations

Our study has several limitations, most notably its observational design and associated risk of residual confounding, despite efforts to account for clinically relevant factors associated with both blood glucose control and HCC risk. The VHA sample is made up largely of middle-aged and older men and is not representative of the United States or global population affected by cirrhosis and at risk of HCC. Due to limited data regarding the cause of death, we were not able to evaluate cause-specific mortality models, though this is an important area of future inquiry. The definitions of MASLD and MetALD have not yet been validated in VHA data. We did not comprehensively evaluate the associations between diabetes medications and HCC risk, which are the subject of future studies by our team.

## CONCLUSIONS

Diabetes and inadequate blood glucose control are independently associated with HCC in a sample of veterans with established cirrhosis due to alcohol, MASLD, and HBV, but not HCV when accounting for HCV treatment status. The risk of HCC associated with diabetes in alcohol-associated cirrhosis was similar to that observed in MASLD cirrhosis. Those with new-onset diabetes after cirrhosis may be at particularly high risk of developing HCC. The standard ADA definition of blood glucose control (Hgb A1c < 7%) may not be the most relevant when assessing HCC risk in diabetes and cirrhosis, and utilizing a higher threshold in this population may be adequate to confer protection against HCC. In the context of the growing prevalence of steatotic liver diseases, these findings can inform clinical practices around blood glucose control for HCC risk modification in cirrhosis.

## Supplementary Material
