# Supplementary material for: HCC is associated with diabetes and longitudinal blood glucose control in a national cohort with cirrhosis
Source: Hepatol Commun. 2023 Dec 7;7(12):e0344. doi: 10.1097/HC9.0000000000000344 (PMC10984661; doi:10.1097/HC9.0000000000000344)
Supplement: Supplementary file 1 [file hc9-7-e0344-s001.docx]

**Supplemental Materials**

**Supplement Table S1. Prevalence of diabetes by etiology of cirrhosis**

|  | **Diabetes** | **No diabetes** |
| --- | --- | --- |
| **Alcohol** | 14471 (55.1%) | 11817 (44.9%) |
| **Alcohol + HCV** | 8977 (51.2%) | 8572 (48.9%) |
| **HBV** | 440 (56.9%) | 334 (43.1%) |
| **HCV** | 8920 (52.2%) | 8166 (47.8%) |
| **MASLD** | 13638 (76.0%) | 4302 (24.0%) |
| **Other** | 1057 (46.6%) | 1213 (53.4%) |

Chi-squared 3180.8, p<0.0001

**Supplement Table S2. Sensitivity analysis excluding individuals with diabetes without at least 50% of follow-up with an available A1c measurement**

|  | **Total Sample** | | **Diabetes at baseline** | |
| --- | --- | --- | --- | --- |
| **Predictor** | **SHR** | **95% CI** | **SHR** | **95% CI** |
| Diabetes | 1.24 | 1.17 – 1.30 |  |  |
| Duration of diabetes  10+ years before cirrhosis  5-10 years before cirrhosis  2-5 years before cirrhosis  0-2 years before cirrhosis |  |  | Ref  1.05  1.13  1.31 | 0.96 – 1.15  1.01 – 1.26  1.18 – 1.46 |
| Time since insulin started  Insulin not started during follow-up  5+ years  2-5 years  0-2 years |  |  | Ref  0.81  0.79  0.79 | 0.71 – 0.92  0.70 – 0.91  0.71 – 0.88 |
| Percent of follow-up with HgbA1c > 7%  (per 10% increase) |  |  | 1.03 | 1.02 – 1.04 |
| Etiology of cirrhosis  MASLD  Alcohol  Alcohol/HCV  HCV  HBV  Other | Ref  1.25  2.85  2.87  1.36  1.06 | 1.14 – 1.37  2.42 – 3.51  2.45 – 3.35  1.04 – 1.18  0.88 – 1.28 | Ref  1.33  2.69  2.68  1.52  0.98 | 1.19 – 1.48  2.14 – 3.38  2.14 – 3.34  1.08 – 2.13  0.74 – 1.30 |
| HCV treatment status  Non-HCV cirrhosis  HCV treated by DAA, cleared  HCV cleared (non-DAA)  HCV treated by DAA, not cleared  HCV not treated, not cleared | Ref  0.12  0.05  no est  2.44 | 0.10 – 0.15  0.03 – 0.07  no est  2.12 – 2.82 | Ref  0.13  0.06  no est  2.31 | 0.10 – 0.16  0.03 – 0.10  no est  1.87 – 2.85 |
| Baseline CTP class  A  B  C | Ref  0.61  0.46 | 0.57 – 0.64  0.39 – 0.53 | Ref  0.66  0.55 | 0.61 – 0.72  0.45 – 0.69 |
| AUDIT-C  Low risk  Moderate risk  High risk  Severe risk | Ref  0.93  0.89  0.76 | 0.85 – 1.01  0.79 – 1.01  0.70 – 0.83 | Ref  1.00  0.89  0.70 | 0.88 – 1.13  0.74 – 1.07  0.61 – 0.81 |
| Age | 1.01 | 1.01 – 1.01 | 1.01 | 1.01 – 1.01 |
| Male sex | 1.87 | 1.53 – 2.30 | 2.00 | 1.50 – 2.67 |
| Race/ethnicity  Non-Hispanic White  Black  Asian  Hispanic  Native American  Other | Ref  0.95  1.26  1.08  1.14  0.84 | 0.89 – 1.01  1.04 – 1.52  0.99 – 1.17  0.93 – 1.41  0.76 – 0.92 | Ref  1.02  1.43  1.18  1.18  0.93 | 0.93 – 1.10  1.11 – 1.85  1.06 – 1.31  0.90 – 1.55  0.82 – 1.05 |
| BMI | 1.01 | 1.01 – 1.01 | 1.01 | 1.01 – 1.02 |
| Tobacco use  Never  Former  Current | Ref  1.05  1.02 | 0.99 – 1.11  0.96 – 1.09 | Ref  1.02  0.90 | 0.95 – 1.10  0.83 – 0.98 |
| Annual Primary Care visits | 0.99 | 0.98 – 1.00 | 0.99 | 0.99 – 1.00 |
| Annual GI/Hepatology visits | 1.02 | 1.02 – 1.03 | 1.01 | 1.00 – 1.02 |

**Supplement Table S3. Sensitivity analysis excluding individuals with no abdominal imaging within 2 years of end of follow-up, total sample**

|  | **Total Sample** | | **Diabetes at baseline** | |
| --- | --- | --- | --- | --- |
| **Predictor** | **SHR** | **95% CI** | **SHR** | **95% CI** |
| Diabetes | 1.14 | 1.08 – 1.19 |  |  |
| Duration of diabetes  10+ years before cirrhosis  5-10 years before cirrhosis  2-5 years before cirrhosis  0-2 years before cirrhosis |  |  | Ref  1.09  1.17  1.34 | 1.00 – 1.19  1.05 – 1.29  1.21 – 1.48 |
| Time since insulin started  Insulin not started during follow-up  5+ years  2-5 years  0-2 years |  |  | Ref  0.80  0.77  0.79 | 0.71 – 0.92  0.68 – 0.88  0.72 – 0.88 |
| Percent of follow-up with HgbA1c > 7%  (per 10% increase) |  |  | 1.04 | 1.03 – 1.05 |

**Supplement Table S4. Baseline characteristics by outcome**

|  | **Total**  **n=81,907** | **Death**  **n=41,215** | **HCC**  **n=8,002** | **Censored**  **n=32,690** | **P** |
| --- | --- | --- | --- | --- | --- |
| Diabetes | 58.0% | 61.7% | 59.1% | 53.1% | <0.001 |
| Etiology of cirrhosis  Alcohol  Alcohol/HCV  HCV  MASLD  HBV  Other | 32.1%  21.4%  20.9%  21.9%  0.9%  2.8% | 39.2%  20.5%  13.2%  23.6%  0.8%  2.7% | 19.1%  38.2% 27.7%  12.4%  0.9%  1.7% | 26.4%  18.5%  28.8%  22.1%  1.2%  3.1% | <0.001 |
| Baseline CTP class  A  B  C | 61.1%  32.3%  6.6% | 44.9%  44.7%  10.4% | 70.4%  26.1%  3.5% | 79.3%  18.1%  2.7% | <0.001 |
| Baseline age (years)  Mean (SD) | 63.0 (8.9) | 64.4 (9.6) | 61.8 (6.6) | 61.7 (8.3) | <0.001 |
