# Supplementary figures and images for: HCC is associated with diabetes and longitudinal blood glucose control in a national cohort with cirrhosis
Source: Hepatol Commun. 2023 Dec 7;7(12):e0344. doi: 10.1097/HC9.0000000000000344 (PMC10984661; doi:10.1097/HC9.0000000000000344)

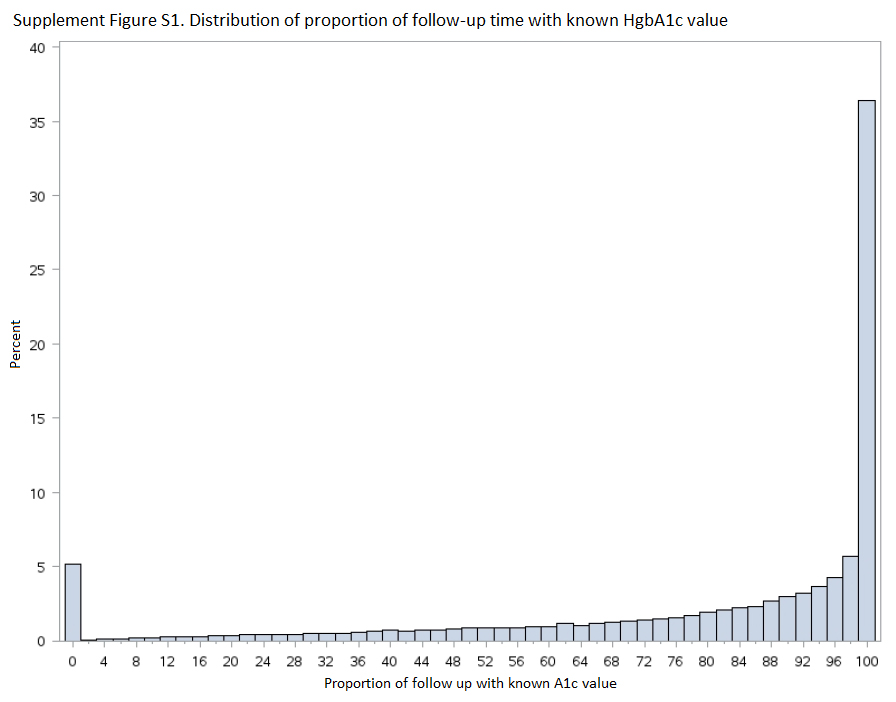

Supplement: Supplementary file 2 [file hc9-7-e0344-s002.jpg]
